# Retraction: Stein, J.A. et al. The Effects of Acute Caffeine Supplementation on Performance in Trained CrossFit Athletes. *Sports* 2019, *7*, 95

**DOI:** 10.3390/sports8020024

**Published:** 2020-02-18

**Authors:** Jesse A. Stein, Melitza Ramirez, Katie M. Heinrich

**Affiliations:** Department of Kinesiology, College of Health and Human Sciences, Kansas State University, Manhattan, KS 66506, USA; melitzar@ksu.edu (M.R.); kmhphd@ksu.edu (K.M.H.)

All authors of the published article [1] have agreed to retract it based on the basis of a data entry error (Figure 1). After re-examination of our results for our published manuscript in *Sports*, we identified incorrectly entered data for six subjects on two variables. Specifically, the subjects who received caffeine during the first session and placebo during the second session (Session 1_Treatment = 2) were incorrectly recorded in the ‘Reps_Placebo’ and ‘Reps_Caffeine’ columns. Thus, the statistical results in our published manuscript were in inaccurate regarding caffeine’s effect during CrossFit exercise. Upon correction and re-analysis, we found that rather than a significant effect of caffeine, there was a significant effect of time (i.e., a learning effect). The paper [1] will therefore be retracted. We apologize to the readers of *Sports* for any inconvenience caused.

## Figures and Tables

**Figure 1:**
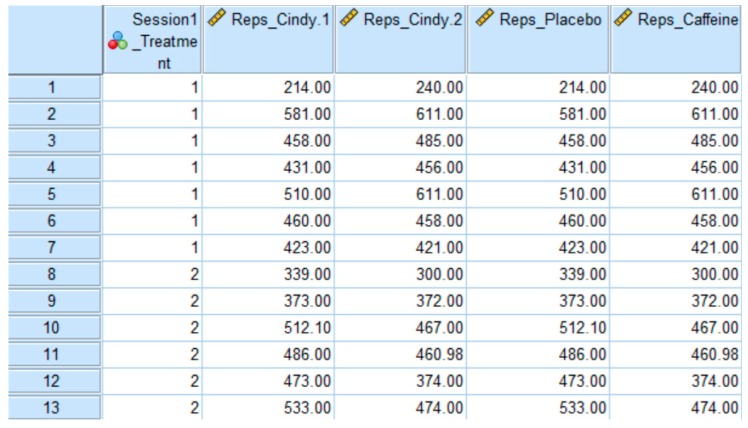
Database for [1]. Each row represents a subject in our investigation. Session 1_Treatment represents the treatment order (1 = placebo then caffeine, 2 = caffeine then placebo). Reps_Cindy.1 and Reps_Cindy.2 represent the total number of repetitions performed during session 1 and session 2, respectively. Reps_Placebo and Reps_Caffeine represent the total number of repetitions performed during the placebo and caffeine sessions, respectively.
